# Suggested Measures for the Regulation of Prostitution and the Repression of Syphilis

**Published:** 1881-09

**Authors:** 


### SUGGESTED MEASURES FOR THE REGULATION OF PROSTITUTION AND THE REPRESSION OF SYPHILIS.

Dr. Casey A. Wood, M. D., Physician to the Woman's Hospital, Montreal, offers (*Canada Medical Record*) the following temperate suggestions for the regulation of prostitution and the repression of syphilis :

Physicians should have power to communicate to the chief of police the names of those prostitutes from whom any of their patients has contracted disease. The medical man should satisfy himself that the patient is in a position to state positively when, where, and from whom, he caught the contagion, and that the female is in the habit of distributing her favors promiscuously or for money. Where there is any doubt about the last two points the suspected woman should have the benefit of it, but in the majority of instances the police would be able to settle the question satisfactorily. Having satisfied himself on these points the chief should have power to serve a notice on the woman to forward to him, within twenty-four hours, a certificate from a regular practitioner, of her being in a healthy state, or else, if she be a common prostitute, to present herself at the hospital for treatment. In case of those who are not "common," in the ordinary acceptation of the term, *i. e.*, who do not practice their trade openly, and do not live in brothels, it would be justifiable to accept a certificate from a regalar practitioner that the woman is under treatment by him, and that he would use every means in his power to prevent her from cohabiting until she recovered. In this way (for all these proceedings would be kept secret, and neither the name of the male sufferer nor of the female patient would be divulged) scandal would be prevented in the case of occasional and otherwise "respectable female."

For the other class, those who are generally recognized strumpets, neglect or refusal to furnish a proper certificate, or to undergo treatment, if diseased, would justify their arrest and forcible detention in special wards of the hospital for a time discretionary with the officials in charge. Action of this kind would encourage the voluntary system and leave coercion as a *dernier ressort.* It would incite women to apply for treatment at once, and not wait until they were compelled to quarantine themselves by the strong arm of the law.

It would respect the respectable, but punish the guilty. Voluntary patients might be allowed to leave the hospital when they desired, but they should be warned that any attempt to return to their trade until fully cured would involve their semi-imprisonment in the "coercion" wards of the hospital, and cut them off from all the privileges of the voluntary side. Examinations should be made voluntary in a dispensary attached to the hospital and a small fee (in Hamburgh, where the regulation system is in vogue, it is only a mark) should be charged. As soon as the intention of periodical examinations was known they would begin to be appreciated, and, in time, the great majority of the prostitutes in the city would be likely to present themselves for medical inspection. A larger fee might be charged for attending prostitutes at their houses. Certificates of good health might be issued if asked for by the women, but it must be understood that they are not considered necessary. It would, of course, be out of the question to admit students to any part of the hospital except to the coercion wards. This portion of the institution, being in some sense a city house of correction, would have a good claim for civic support, and in that case might be overlooked by a local inspector. In the event of a hardened sinner persisting in spreading venereal diseases instead of applying to hospital for relief, and necessitating repeated arrests, it would be justifiable to have her registered and examined by the medical officer not less frequently than once a week. This would be a greater punishment to her, in view of the treatment of her other sisters in vice, than imprisonment.

To complete these suggested regulations, it ought to be made possible for an inmate of a house of ill fame to abandon her life of infamy free of any claim for board, liquors, clothes, etc., the brothel-keeper may have upon her. It is, of course, to the interest of procurers and keepers to exert as great an influence upon their stock-in-trade as possible, and for this purpose many of them try to keep the girls in debt, so that they are compelled to continue in their old ways. It would be a good idea also to subject brothel-keepers to a heavy fine, if it be proved that they allow any of their women to remain in their houses after becoming diseased. The proceeds of such fines would go to defray the expenses of the hospital.

The advantages of the measures above specified recommend themselves because: (1) the legislation involved is not a one-sided treatment of woman as if she were made for man simply to gratify his lust upon; (2) they leave a way open to those erring ones who desire to reform; (3) women are not compelled, except as a last resort, to undergo a degrading periodical examination by public officers; (4) the system does not condemn to a life of hopeless infamy those who err temporarily, or who are seduced by designing men; (5) they provide for clandestine prostitution; (6) they are voluntary to a very great degree, and attempt to do by kindness what coercion has, over and over again, failed to accomplish; and, lastly (7) they do not violate the sanctity of private houses, as the system of forced registration is sure to do.

